# Supplementary material for: Continentality determines warming or cooling impact of heavy rainfall events on permafrost
Source: Nat Commun. 2023 Jun 16;14:3578. doi: 10.1038/s41467-023-39325-4 (PMC10275877; doi:10.1038/s41467-023-39325-4)
Supplement: Supplementary file 1 — Supplementary information [file 41467_2023_39325_MOESM1_ESM.pdf]

Continentality determines warming or cooling impact of heavy rainfall events on permafrost

Alexandra Hamm, Rúna Í. Magnússon, Ahmad Jan Khattak, and Andrew Frampton

Supplementary Information

In the Supplementary Information, we describe additional information about the model simulations (section 1), the literature search and analysis (section 2), the sensitivity analysis (section 3), additional analysis on multi-year effects (section 4), and present additional Figures (section 5) and Tables (section 6).

Index

1 Literature review and analysis
  1.1 Literature Search
  1.2 Statistical Analysis
2 Model Parameters and Forcing
3 Model Sensitivity Analysis
  3.1 Uniform Rainfall Distribution
  3.2 Heavy rain event magnitude
  3.3 Multiyear effects
  3.4 Soil Stratigraphy
4 Supplementary Figures
  Figures S1 to S17
5 Supplementary Tables
  Tables S1 to S5

Supplementary Text

1 Literature Review and Analysis

1.1 Literature Search

Due to the limited number of studies reporting effects of heavy rainfall on the soil thermal regime of permafrost soils at the time of writing, and substantial variability in types of studies and reported effects within existing literature, we conducted a scoping literature review[1]. We adopted a hybrid search strategy to create an overview of relevant literature to date. Our approach consisted of (1) a systematic database search, complemented with purposive literature screening using (2) snowball searching and (3) Google Scholar alerts for recent studies. As a result, we do not consider our literature search to be a fully systematic search. When systematic approaches yield few studies, or studies with substantial variability in methodology and reported variables, a purposive search strategy generally leads to a more complete insight[1]. After hybrid search, we further assessed identified studies for eligibility using PICOS criteria[2]. Occasional exceptions were made for several studies that fell outside the defined scope, but yielded relevant insights into the role of rainfall in in permafrost thermal dynamics (see “Exceptions to PICOS criteria”). All identified studies were screened by two researchers (AH and RM) and discussed until consensus was reached. Due to variability in the reporting of effects sizes, study designs and reported metrics (e.g. thaw depth or soil temperature), we used a vote-counting approach based on a predefined rule set to qualitatively subdivide results of identified studies into warming and cooling effects (see “Synthesizing approach”). We provide brief descriptions of the findings of each study in the synthesis table, to provide a nuanced view of rainfall effects on permafrost soil thermal regime. Lastly, we evaluated whether site climatology differed significantly among sites for which warming and cooling effects were reported (see “2.2 Statistical analysis”).

1.1.1 Systematic database search

We searched the ISI Web of Science Databases using the following search string. We restricted the search to peer reviewed articles. The search results are available from https://www.webofscience.com/wos/woscc/summary/6aba447e-3966-4902-b3ed-980113eaae8d-6d2188c7/relevance/1 (last accessed on 16-02-2023). The used search string was:

"permafrost" AND "increas* rain" OR "increas* summer precipitation" OR "increas* rainfall" OR "heavy rain" OR "heavy rainfall" OR "heavy summer precipitation" OR "extreme rain" OR "extreme rainfall" OR "extreme summer precipitation" OR "wet summer" OR "precipitation experiment" OR "rain experiment" OR "rainfall experiment" OR "precipitation manipulation" OR "rain manipulation" OR "rainfall manipulation" OR "irrigation" AND "ground temperature*" OR "soil temperature*" OR "thaw depth*" OR "active layer thickness" OR "active layer depth" OR "active layer"

1.1.2 Purposive search

Due to the limited body of literature reporting on effects of increased rainfall on the thermal regime of permafrost soils, we extended our literature search with a purposive search. Two researchers set Google Scholar alerts and screened results for eligibility (RM: keywords "permafrost thermokarst rain vegetation", period May 2022 – February 2023, AH: keywords "permafrost modelling" and "permafrost rain OR rainfall", period February 2021 – February 2023). We additionally used a non-systematic version of "backward snowballing"[3], in which we retrieved studies cited in literature identified thus far (i.e. through systematic search, Google Scholar alerts and earlier rounds of snowball searching). For the purposes of this study, we focused on effects of heavy rainfall events on ground temperatures observed in summer (June – August) within the same year. Effects of heavy rainfall events may persist for multiple years[4], but few studies report the duration of effects, restricting our inventory to same year effects.

1.1.3 PICOS Criteria

In order to provide an overview of the current state of knowledge on the effects of heavy rainfall on permafrost stability, we selected studies according to the following criteria, presented as a PICOS Statement[2]. We set the following PICOS criteria for inclusion into literature review:

1. Population: studies reporting on changes in soil thermal dynamics under increased rainfall, in sites underlain by permafrost.
2. Interventions: experimentally increased rainfall (e.g. irrigation), modeled increases in rainfall or natural annual variability in rainfall
3. Comparators: control treatments with ambient rainfall, simulations with baseline rainfall or natural annual variability in rainfall
4. Outcome: studies reporting effects on soil temperature, thaw depth or active layer thickness
5. Study design: monitoring, experimental or modeling studies

1.1.4 Exceptions to PICOS Criteria

In a few cases, we retained studies in the synthesis table that were formally outside the defined PICOS criteria. Firstly, we include a study by Göckede et al. [5], who investigate the effects of a drainage intervention rather than an increase in rainfall. This resulted in a wetter and drier site, for which extensive monitoring data are reported. Since this provides a valuable insight into changes in soil thermal behavior under wetting/drying, we decided to include the study into the literature review and discussion. Similarly, we include a study by Clayton et al. [6] comparing active layer thickness across natural soil moisture gradients. While not testing effects of rainfall per se, this study provides similar detailed insight into the role of soil wetting in permafrost thaw for a large number of site across a

large number of sites across the Alaskan Arctic. Lastly, we included results from Iijima et al. [7], who report changes to the soil thermal regime in a summer following a combination of increased pre-winter rainfall and winter snowfall. Although these are not same-season effects, it provides additional insight into potential carry-over effects of late summer rainfall into next season, and potential effects of combined increases in rainfall and snowfall. These studies are included in the synthesis table and discussion in the main manuscript, but not into the statistical evaluation of warming and cooling effects of heavy rainfall across the Arctic (see section 2.2).

1.2 Statistical Analysis

1.2.1 Vote-counting Procedure

In order to synthesize the identified literature, we adopted a two-fold procedure. First, we provide a summary of the literature, reporting authors and year of publication, location, study period, the main method applied as well as the main effect observed, and the main processes that the observed effect can be attributed to (Table S1). Wherever quantified, we note the effect extent, reported observation depths, as well as the magnitude of the heavy rainfall, and further categorize the studies into irrigation experiments and observed natural variability studies (Table S2). Due to differences in reported metrics, depth and duration of soil temperature observation and differences in rainfall variability and treatments, a formal meta-analysis of effect sizes from synthesized studies was not possible. Hence, we adopt a vote counting procedure Bushman and Wang [8] based on a rule set described below. Using this rule set, we subdivide reported changes in thaw depth, active layer thickness and soil temperatures into ”warming” effect, ”cooling” effect, ”no effect” or ”both” effects.

- Since not all studies report legacy effects of rainfall events, we evaluated only same-season responses of active layer thickness, thaw depths and soil temperatures in summer (June-September).
- We judged that active layer thickness, thaw depths and soil temperatures are progressively less indicative of permafrost degradation. Hence, in studies that report multiple metrics, we focus on reported effects on active layer thickness or thaw depth.
- Field-observed increases or decreases in active layer thickness, thaw depth or soil temperature under increased rainfall or rainfall manipulation is counted as a ”warming effect” or ”cooling effect”, respectively.
- If provided, the magnitude of rainfall events and interventions, as well as the magnitude of observed changes in soil temperature, thaw depth or active layer thickness are provided.
- If the study reports that no increase or decrease in any of the considered metrics could be observed based on rainfall treatment or rainfall events, the study is listed as ”no effect”.
- If the study reports diverging effects based on timing within the season, sub-plot within a site, or the magnitude of a rainfall event or treatment, the study is assigned to the ”both effects” category. We include the source of heterogeneity in response as a comment.
- If the study reports diverging warming or cooling effects across soil depth, we report the effect at the depth that is situated closest to the thaw front and assign a warming / cooling effect accordingly. We include a comment in the synthesis table that effects diverged over soil depth.

The result of this vote-counting procedure was added to the synthesis tables (Table S1 and S2).

1.2.2 Site Selection for Statistical Analysis

Secondly, we analyzed how the vote-counting outcomes varied across site climatology. We expected that the effect of heavy rainfall on the soil thermal regime of permafrost soils would vary among climatic regions, and would depend on the extent to which alterations of soil hydrothermal properties changed the response of soil temperatures to

outside air temperatures (see ”Introduction” in main text). To investigate these expectations, we compared outcomes of the vote-counting procedure to site temperature and precipitation data. We selected only those studies that report field-measured, spatially explicit responses of soil temperature, thaw depth or active layer thickness under experimental rainfall increases or natural annual variability in rainfall (see ”PICOS Criteria” below), and omitted studies not meeting those criteria[9,10,11,12,13] We also exclude studies reporting results of other interventions than rainfall that were included in the literature review as supporting evidence[5,6] and studies reporting only next-season effects of heavy rainfall events[7], see ”Exceptions to PICOS criteria”.

1. Population: field studies reporting on changes in soil thermal dynamics under increased rainfall, in sites underlain by permafrost with known locations (coordinates are reported in the study)
2. Interventions: experimentally increased rainfall (e.g. irrigation) or natural variability in rainfall
3. Comparators: control treatments with ambient rainfall or natural annual variability in rainfall
4. Outcome: studies reporting first-year effects on soil temperature, thaw depth or active layer thickness
5. Study design: monitoring and experimental studies reporting in situ field-measured data

One study[14] reports a single observed effect for a set of 10 subsites that were up to 550 km apart with two subgroups "north" and "within/south" of the Tanggula mountain range. Due to the large spatial distance between the two sub-locations and the presence of the mountain range, we split these results into two individual groups each reporting their individual climatic conditions and continentality value. Other studies report effects for single monitoring or experimental sites, or report a single observed outcome for subsites or experimental plots situated in close proximity (up to 10 km) to each other. Due to the close proximity of such within-study subsites relative to the spatial resolution of the climate reanalysis data ($\sim$30 km), and the fact that most of these studies report overall effects of rainfall across such subsites, these studies were treated as single data points, reporting the study’s overall observed effect. From this selection, 14 studies remained (see PRISMA workflow in Fig. S16).

1.2.3 Site Climate Data

For these sites, we extracted monthly temperatures and precipitation data from ERA5 reanalysis data for a common and recent period (1991–2020)[15] to calculate average temperature and total precipitation in winter (December––February), spring (March—May), summer (June—August) and fall (September-–November). Apart from seasonally averaged temperature and precipitation data, we calculated Conrad’s Continentality Index (CCI) for each site over the same period from the same ERA5 data. Conrad’s continentality index characterizes a place as "oceanic" to "continental" based on the maximum and minimum annual temperature and the latitude at which the place is located (Eq. 1 in the main text).

1.2.4 Statistical Analysis

This resulting dataset of 14 sites with observed rainfall effect, coordinates and climate data was used to assess patterns among reported effects on the soil thermal regime (warming, cooling, both or no effect) and site climatological data (total precipitation and mean temperature for winter, spring, summer and fall, and CCI). We then assessed whether different responses were associated with contrasts in site climatology using a combination of exploratory (PCA) and inferential (Wilcoxon rank sum test) statistics. First, we ran a PCA on site climatological data (averaged temperatures and seasonal total precipitation per season and CCI) and visually assessed patterns of reported effects of heavy rainfall on the soil thermal regime against the first two principal components using a biplot. Since the biplot showed a strong alignment of the “cooling” and “warming” sites with CCI and winter precipitation (Fig. 2a), we tested whether CCI and winter precipitation differed significantly among sites that were assigned to different response classes during the vote-counting procedure (warming: $n = 7$, cooling: $n = 6$, none: $n = 1$, both: $n = 0$). We used non-parametric tests to account for relatively small sample sizes. Due to the low number of observations in the “both” and “no-effect”

classes, we only tested for differences among the "warming" and "cooling" classes, using a Wilcoxon rank sum test and visualized results using boxplots. We assessed potential violation of spatially independent residuals using Moran's I. We set our significance criterium at $\alpha = 0.05$.

2 Model Parameters and Forcing

In ATS, soil physical parameters are user defined. A list of parameter values used in this study can be found in Table S4. In our study we focus on generalizations of climatic conditions combined with generalizations of soil types, considering representation of organic and mineral soil textures with thermal and hydraulic parameters based on literature values. For cases where site-specific data is available, soil parameters can be calibrated against measurements to represent local site conditions. In our study however, we focus on larger-scale climatological contrasts. We do not aim to accurately resolve site-specific conditions, but rather obtain a general idea of the system behavior for class-type representations of organic and mineral soils.

ATS is further driven by a hydro-meteorological forcing dataset consisting of air temperature, incoming shortwave radiation, windspeed, relative humidity, and rain- and snowfall at a desired timestep. A representation of the forcing data in our four climate scenario simulations can be found in Figure S1. While incoming shortwave radiation, windspeed and relative humidity are being held constant between the climate scenarios, temperature either reflects a continental or maritime characterized climate (see methods section in the main text). Water enters the model domain through precipitation, which is defined as rain when air temperatures are above 0°C and as snow if air temperatures reach 0°C and below. This causes a slight temporal shift between the different climate cases in the snow and rain period, respectively. In ATS, rain temperature is assumed to be equal to air temperature, which is a reasonable assumption[16] given the complex nature of meteorological conditions. Water can leave the model domain via evaporation. This process only accounts for bare-ground evaporation and does not explicitly resolve transpiration by plants.

Rain water that does not evaporate, infiltrates into the soil. The relationship between precipitation and soil moisture is governed by the hydraulic conductivity of the soil, which in turn is influenced by permeability, porosity, and water retention characteristics. The model accounts for saturation-dependent thermal conductivity by calculating the fraction of pore space occupied by ice, unfrozen water and air. In essence, the bulk soil thermal conductivity depends on the phase saturation of the pore-space filling fluid. Unfrozen water content follows classical soil moisture retention curves, using the van Genuchten formulation, and is combined with the Clausius-Clapeyron relation and latent heat of fusion to obtain frozen water fractions[17]. This leads to a non-linear coupling between soil moisture and soil temperature. Thus, a dry soil can greatly increase its bulk thermal conductivity by addition of water, however, if the soil is already wet and near saturation, then the addition of water might only cause a small increase before reaching saturation.

3 Model Sensitivity Analysis

To assess the sensitivity of our model experiment to quantities such as rainfall intensity and organic layer thickness (soil stratigraphy), we created additional sensitivity analysis model scenarios. In one scenario, we vary the thickness of the overlaying organic layer to evaluate how soil physical properties change the simulated effect, and in a three scenarios we change the magnitude and distribution of additional precipitation.

3.1 Uniform Rainfall Distribution

In the original scenario described in the main text, additional summer rainfall is added in three distinct heavy rainfall events representing precipitation events with a 100-year recurrence interval in observed weather station data throughout permafrost environments (see Table 1 in the main text). The recurrence interval is based on the available data and covers between seven and 20 years of the most recent observations. Hence, the recurrence interval is based on current climatic conditions and might be subject to change due to ongoing climate change.

To assess the influence of a more uniform increase in precipitation, we performed a set of simulations in which total summer precipitation is increased by 50% representing the upper boundary of estimated overall future summer precipitation increase in the Arctic[18]. The result is shown in Figure S12, which represents the same simulation results but with the adjusted precipitation rates as in Figure 3 in the main text. Due to the extended period of increased precipitation, the time axis differs to the one in Figure 3 in the main text. Overall, the effect direction is the same as in the model simulations in the main text with heavy rainfall events. Dry climates show a warming effect, wet climates show an overall cooling by the end of the summer season. However, during early summer, just after air temperatures reached 0°C, an initial warming effect in both, top and subsoil in all climate cases can be observed. With the start of the development of the active layer, the wet scenarios then show a cooling effect, while in the dry scenarios the enhanced temperatures further increase. The overall warming effect in the subsoil is lower than in the heavy rain event simulations (up to 0.1°C), while the cooling effect is similar (just over -0.1°C).

3.2 Heavy rain event magnitude

In our original model scenarios, heavy rain events are defined as the 100 year recurrence interval of heavy rain events in June, July, and August. However, the range of uncertainty about future precipitation extremes is significant. Hence, we conducted additional sensitivity analyses and simulated heavy rain events that are 50% less, as well as 50% more intense (Fig. S13).

In the 50% less intense heavy rainfall scenario (Fig. S13a), subsoil temperatures exhibit the same patterns as in the original scenario (see Fig. 3). However, the warming temperature difference effect in the dry climate cases is reduced by ∼0.1°C (∼30%). The cooling effect in the subsoil in the wet climate cases, on the other hand, is reduced by 0.05°C, and thereby only half as strong. In the topsoil, the cooling effect in the wet scenarios is reduced by 50% but the warming in the warm-dry scenario remains at a maximum of 0.2°C. Subsoil warming in this scenario remains strong enough to increase ALT by 2 cm in the dry climate cases just like in the original heavy rainfall scenario. Similarly, no change in ALT is visible in the wet climate cases. Notably, a delay in total freeze-up by the end of the thawing season can not be seen in this scenario, indicating that latent heat requirements have not increased as much as in the original scenario and allow simultaneous freeze-up between the ref. case and the HR case.

In the scenario with 50% more intense heavy rainfall events (Fig. S13b), only a small increase in maximum subsoil warming in the dry climate cases can be seen compared to the original scenario. Similarly, the wet cases show very similar cooling responses as in the original scenario. The topsoil, on the other hand, follows a somewhat linear trend from the 50% reduced, over the original scenario to the 50% increased scenario, where topsoil cooling in the wet climate cases and in the cold-dry climate is weakest in the 50% reduced scenario (up to -0.2°C), and strongest in the 50% increased scenario (up to -0.65°C) with the original scenario located in between (up to -0.45°C). This indicates that the effect of subsoil cooling might level off at a certain rainfall intensity, while topsoil cooling might get intensified in even stronger heavy rainfall events. Thaw depth development in the 50% increased scenario shows that active layer depth is not increased more than in the original scenario (2 cm). In the dry cases but actually shows that thaw depth is enhanced for a short period of time in the wet climate HR cases as well indicating non-linear between rainfall intensities and ALT development. Most notably, total freeze-up is strongly delayed in this scenario (13 and 11 days in the cold-dry and warm-dry, respectively, and 4 and 3 days in the cold-wet and warm-wet scenario, respectively).

Overall, a non-linear effect of ground temperature effects can be seen, especially in the subsoil, from a 50% reduced to a 50% increased heavy rainfall scenario with substantial changes in latent heat requirements leading to delays in freeze-up timing. It is therefore important to pay close attention to future developments of heavy rainfall events and how to present them in models.

3.3 Multiyear effects

In our main results we focus on the same-season effects of heavy rainfall. However, effects of a single or multiple wet summers might persist for several years as for example in Magnússon et al. [[4]]. Studying multi-year effects of heavy summer rainfall is challenging as a set of non-linear effects can change the consequences of a single heavy

rain event drastically. Onset of snowfall, snow cover thickness, and lateral redistribution of water are examples of possible processes that can change the system behavior. We conducted a basic multi-year analysis in which we simulate two scenarios: A single year of heavy summer rainfall events and four consecutive heavy summer rainfall years followed by ten years of average conditions. All variables except for summer precipitation in one and four years, respectively, are being held constant over the simulation period.

In the scenario with a single heavy summer rainfall year, the effect is strongest within the same season in the dry climate cases and shows an opposing effect to what is observed in the same season from the first year after the heavy rainfall event onward (Fig. S14a). The cooling in the dry cases after the year of heavy rainfall is likely related to the increased heat capacity and latent heat requirements in the subsoil after heavy rainfall has caused increased liquid saturation. The effect diminishes over the years as drying from the surface (evaporation) removes the additional water and after ten years, the temperature difference between the ref. case and the HR case is less than 0.1°C. In the wet scenarios, the cooling effect in the same season as the heavy summer rainfall gets enhanced in the second year (the first year after the heavy rainfall summer) and increases to up to $\sim$-0.3°C in the cold-wet and $\sim$-0.25°C in the warm-wet climate case. This suggests that heavy rainfall might exert a stronger influence on ground temperatures in the year after the year with heavy summer rainfall. The rebound to average conditions is faster in the cold-wet than the warm-wet scenario, but after ten years both cases show no significant temperature between the ref. case and the HR case anymore ($<$ 0.1°C).

Four consecutive years of heavy rainfall with additional ten years of average rainfall conditions shows diverging effects depending on the climate case (Fig. S14). In the cold-dry, four years of precipitation seem to shift the system from a dry towards a wet climate effect as the warming effect in late summer diminishes in the fourth year of heavy precipitation and even shows a significant cooling in the early season. In contrast, the warm-dry scenario seems to experience a temporal lag in the onset of warming, but reaches the same amount of late warm-season warming in late August. After ten years of average precipitation the temperature difference in the dry climate cases is still $>$0.1°C. In the wet climates, the initial cooling effect is enhanced after several years of heavy rainfall events (up to $\sim$0.5°C after the fourth year) but experiences more rapid rebound to baseline conditions than the dry climate cases.

3.4 Soil Stratigraphy

Organic layers are a common feature in permafrost landscapes. They are typically between 5 and 30 cm thick and consist of highly organic material[19]. Due to the differences in soil thermal and hydrological properties between organic and mineral soil, the organic layer plays an important role in the climate feedback loop. In our main study, we assume an organic layer of 20 cm thickness. To evaluate the impact of different organic layer thicknesses, we created two additional scenarios. One with a 50 cm thick organic layer and a scenario without any organic layer. Figure S15 shows the temperature response analogous to Figure 3 in the main text for a scenario with (a) a 50 cm thick organic layer and (b) no organic layer. Increasing the organic layer thickness (a) caused a similar system response to the shallower, 20 cm thick organic layer. In the subsoil, both the warming and the cooling effect are enhanced from originally 0.3°C warming and 0.1°C cooling to around 0.35°C warming and 0.2°C cooling. In the topsoil, all scenarios respond with an enhanced cooling to the heavy rainfall events in the thicker organic layer scenario as compared to the original scenario. Topsoils are up to 0.65°C colder just after a heavy rain event as compared to the reference case. In a scenario without any organic layer (b), we also find dry climate scenarios to respond with a warming and the wet scenarios responding with a slight warming in early summer followed by a small net cooling in later summer. The effect is overall smaller than in the original scenario and enhanced organic thickness scenario. In the topsoil, a similar pattern to the subsoil emerges. Dry climates respond with warming first followed by a cooling response to heavy rainfall, while wet climates respond with cooling right after the rainfall event and subsequent warming until the next event. Overall this suggests that the effect direction is not influenced by varying the thickness of the organic layer. The effect size, however, can change based on how much organic material overlays the mineral soil. Thicker organic layers enhance the effect of heavy rainfall events observed in the original scenario, but soils without significant organic layer may show no significant response in deeper soil layers and no clear effect on active layer development and associated carbon release.

4 Supplementary Figures

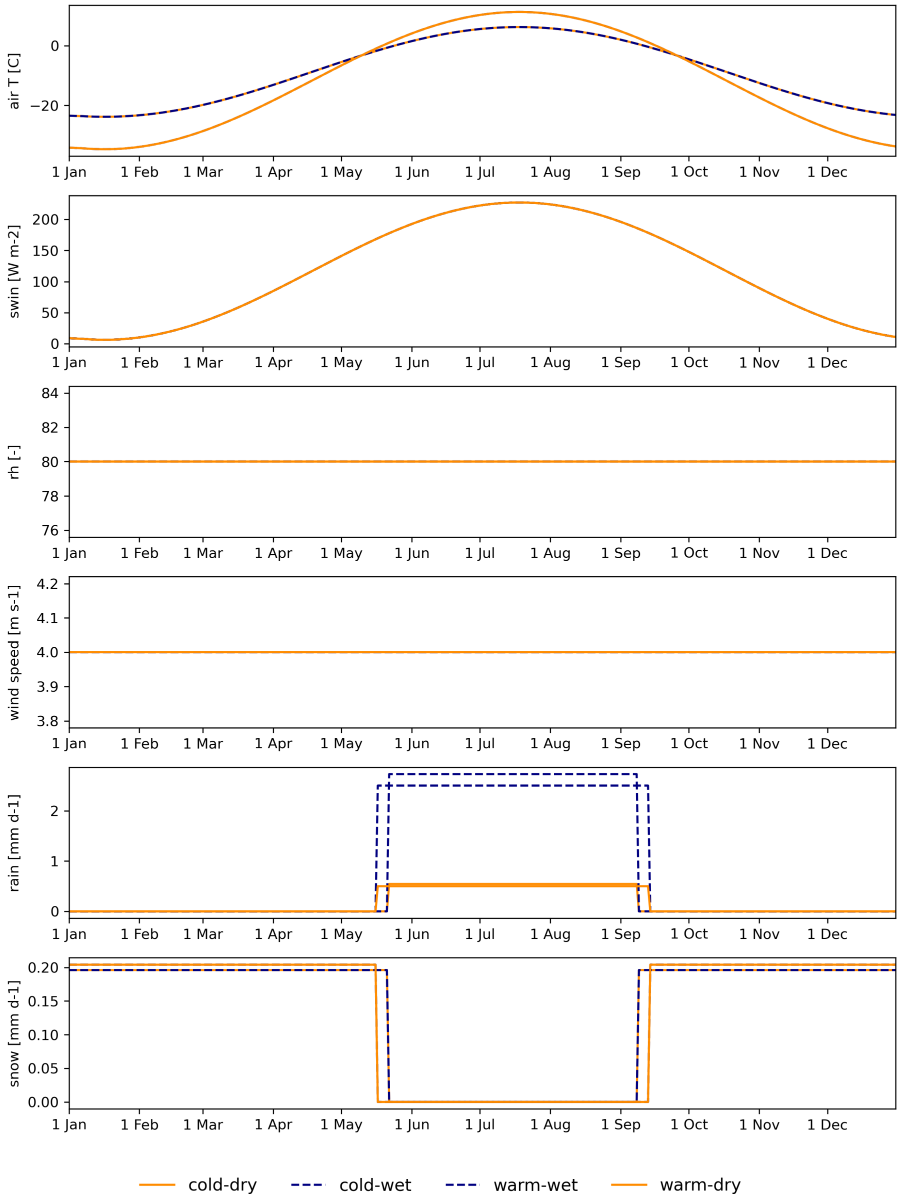

Figure S1: Representation of the forcing variables for the four climate scenarios.

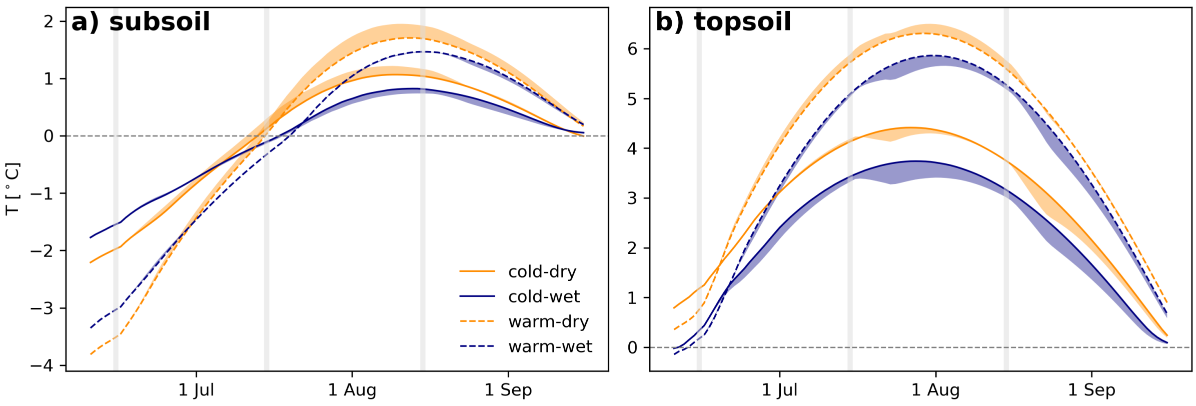

Figure S2: Absolute temperatures in the ref. and heavy rainfall (HR) case from the first heavy rain event until the end of the thawing season in (**a**) the subsoil (75% ALT) and (**b**) the topsoil (25% ALT). Dashed and solid lines represent the ref. case temperatures in each climate scenario, shaded areas indicate the change from ref. to HR case temperature. Daily values are averaged over a 7-day window.

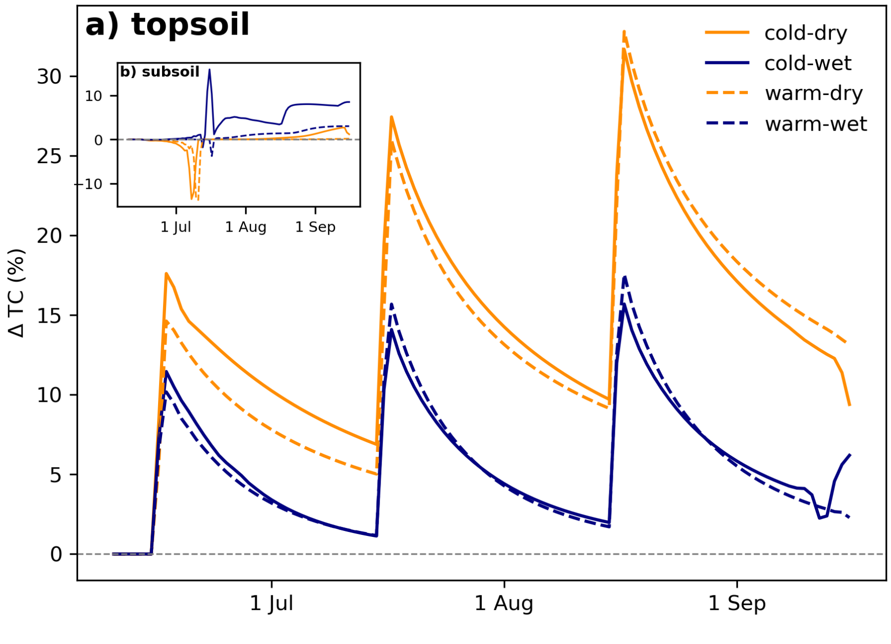

Figure S3: Daily relative difference between the reference case and heavy rainfall (HR) case thermal conductivity in (**a**) the topsoil (25% ALT) and (**b**) the subsoil (75% ALT) in percent.

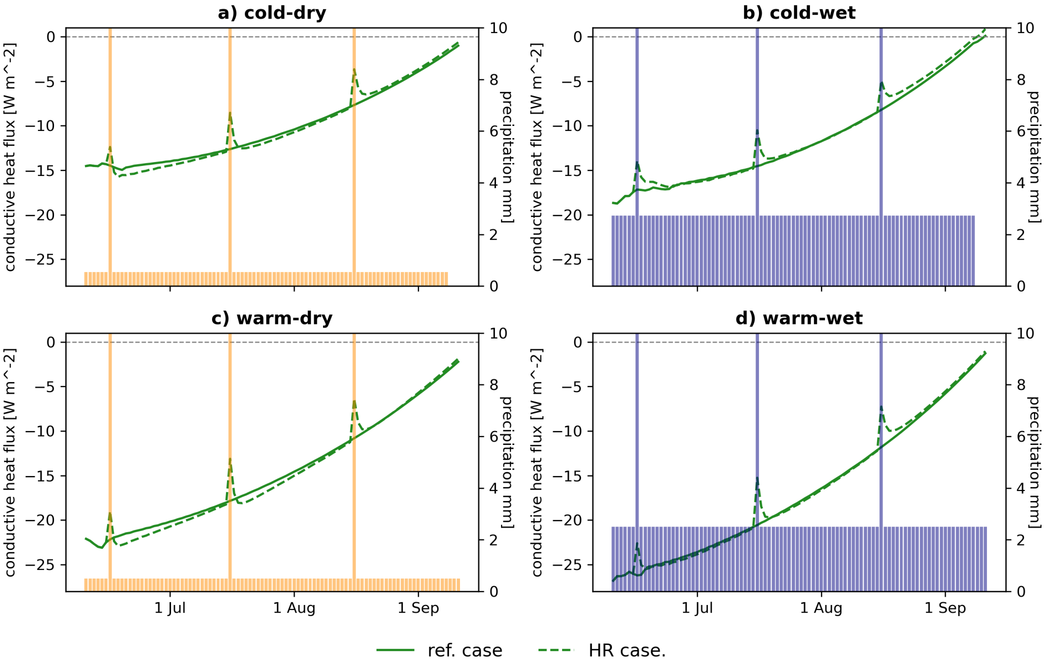

Figure S4: Conductive (diffusive) heat flux in the topsoil (25% ALT) for the reference case (solid green line) and the heavy rainfall case (HR case, dashed green line) during the summer season in (**a**) the cold-dry, (**b**) the cold-wet, (**c**) the warm-dry, and (**d**) the warm-wet case. Negative values indicate heat conduction downwards (heat gain) into the subsurface, positive values indicate heat conduction upwards towards the surface and atmosphere (heat loss). Yellow and blue bars indicate summer rainfall distribution in the dry and wet scenario, respectively. Note that the heavy rainfall events in this plot are capped at 10 mm to enable better comparison between the cases.

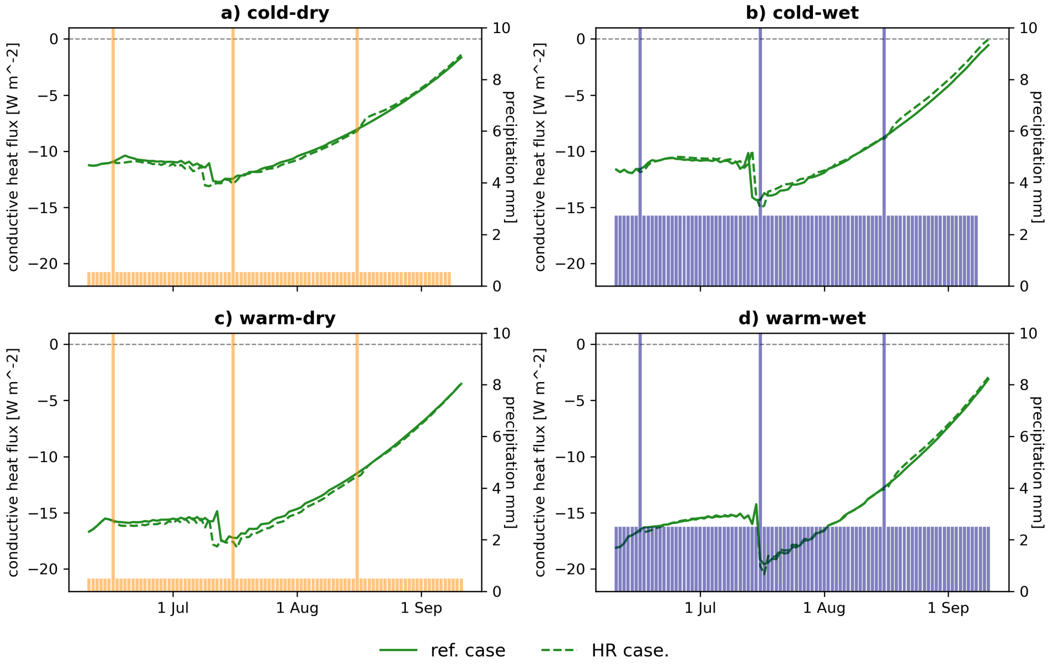

Figure S5: Conductive (diffusive) heat flux in the subsoil (75% ALT) for the ref. case (solid green line) and the heavy rainfall case (HR case, dashed green line) during the summer season in (**a**) the cold-dry, (**b**) the cold-wet, (**c**) the warm-dry, and (**d**) the warm-wet case. Negative values indicate heat conduction downwards (heat gain) into the subsurface, positive values indicate heat conduction upwards towards the surface (heat loss). Yellow and blue bars indicate summer rainfall distribution in the dry and wet scenario, respectively. Note that the heavy rainfall events in this plot are capped at 10 mm to enable better comparison between the cases.

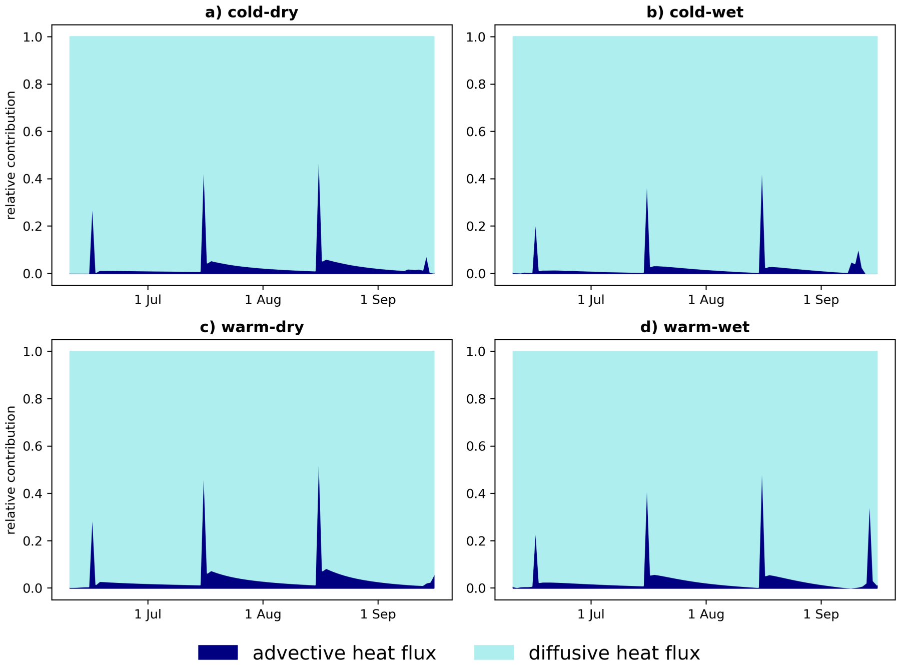

Figure S6: Relative contribution of conductive (diffusive) heat flux (light blue) and advective heat flux (dark blue) to the overall heat flux in the topsoil (25% ALT) in the heavy rainfall (HR) case for (**a**) the cold-dry, (**b**) the cold-wet, (**c**) the warm-dry, and (**d**) the warm-wet case. Contributions are calculated as $R_{\text{adv}} = \frac{Q_{\text{adv}}}{Q_{\text{adv}}+Q_{\text{diff}}}$ for the advective energy contribution and as $R_{\text{diff}} = \frac{Q_{\text{diff}}}{Q_{\text{adv}}+Q_{\text{diff}}}$ for the conductive/diffusive component.

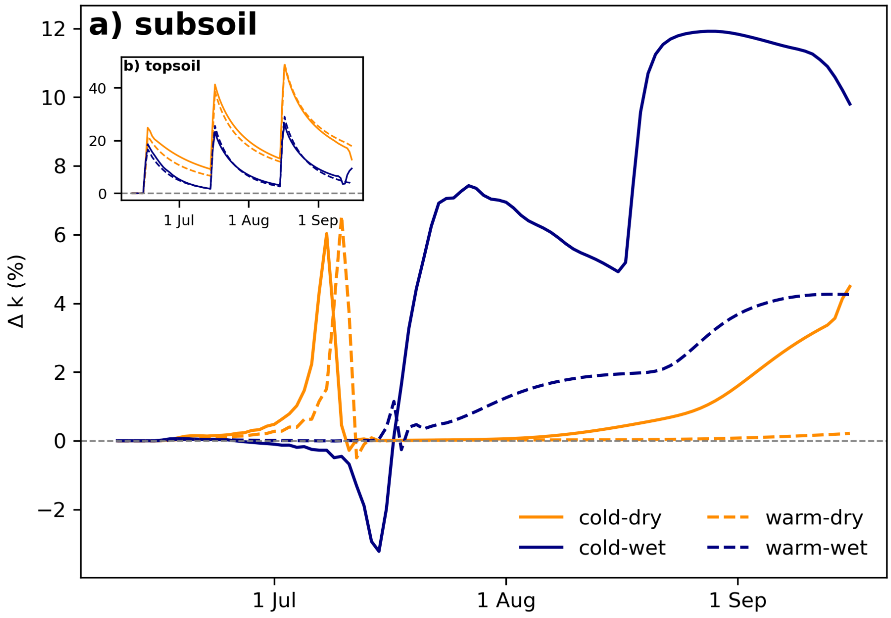

Figure S7: Daily relative difference between the reference case and the heavy rainfall (HR) case heat capacity in (**a**) the subsoil (75% ALT) and (**b**) the topsoil (25% ALT) in percent.

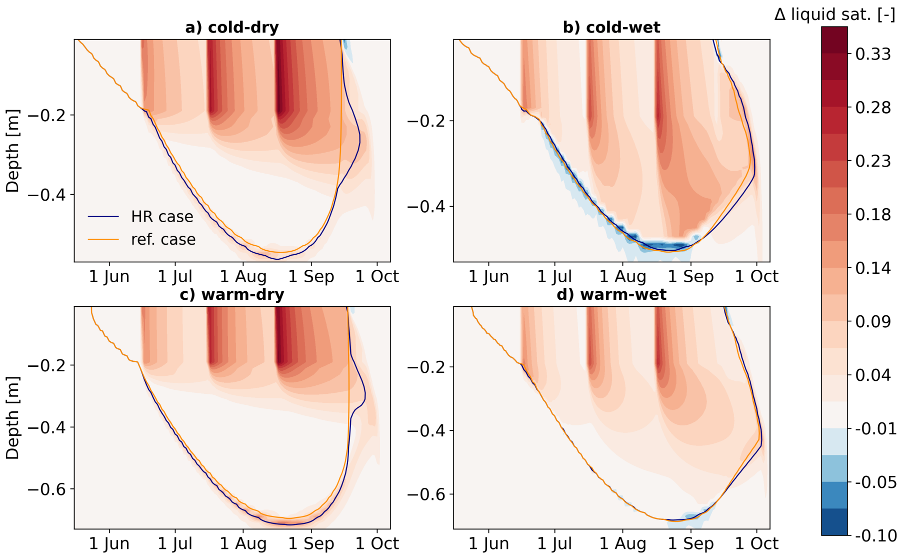

Figure S8: Daily difference between reference case and the heavy rainfall (HR) case (HR–ref. case) liquid saturation throughout the depth profile in (**a**) the cold-dry, (**b**) the cold-wet, (**c**) the warm-dry, and (**d**) the warm-wet case. Shades of red indicate higher liquid saturation in the HR case compared to the reference case, blue shades indicate lower values. The orange and blue contour represent the 0°C isotherm depth in the reference and HR case, respectively.

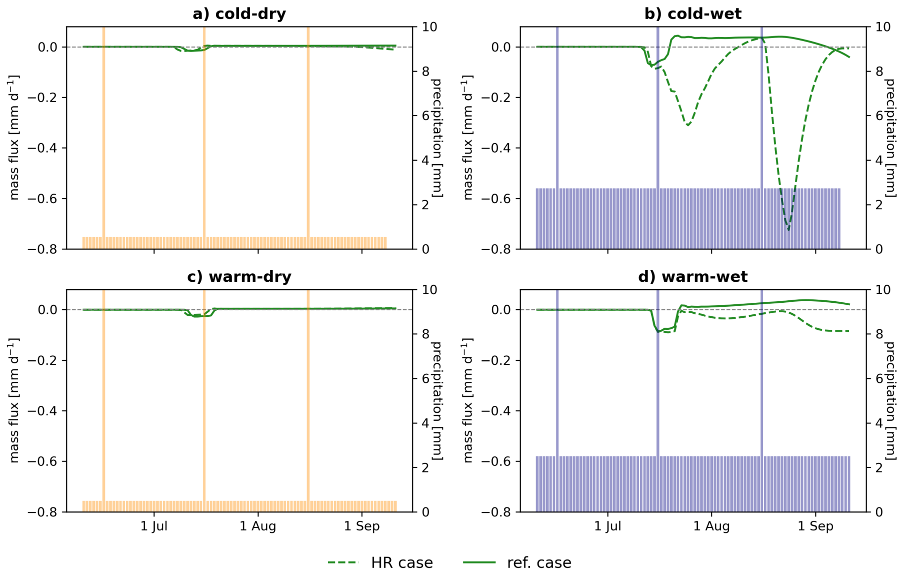

Figure S9: Subsoil (75% ALT) mass flux during the summer season. Solid lines represent the reference case, dashed lines the heavy rainfall (HR) case in (**a**) the cold-dry, (**b**) the cold-wet, (**c**) the warm-dry, and (**d**) the warm-wet case. Negative values indicate downwards mass (water) flux into the deeper layers in the subsurface, positive values indicate upward mass flux towards the surface. Yellow and blue bars indicate summer rainfall distribution in the dry and wet scenario, respectively. Note that the heavy rainfall events in this plot are capped at 10 mm for better visualization.

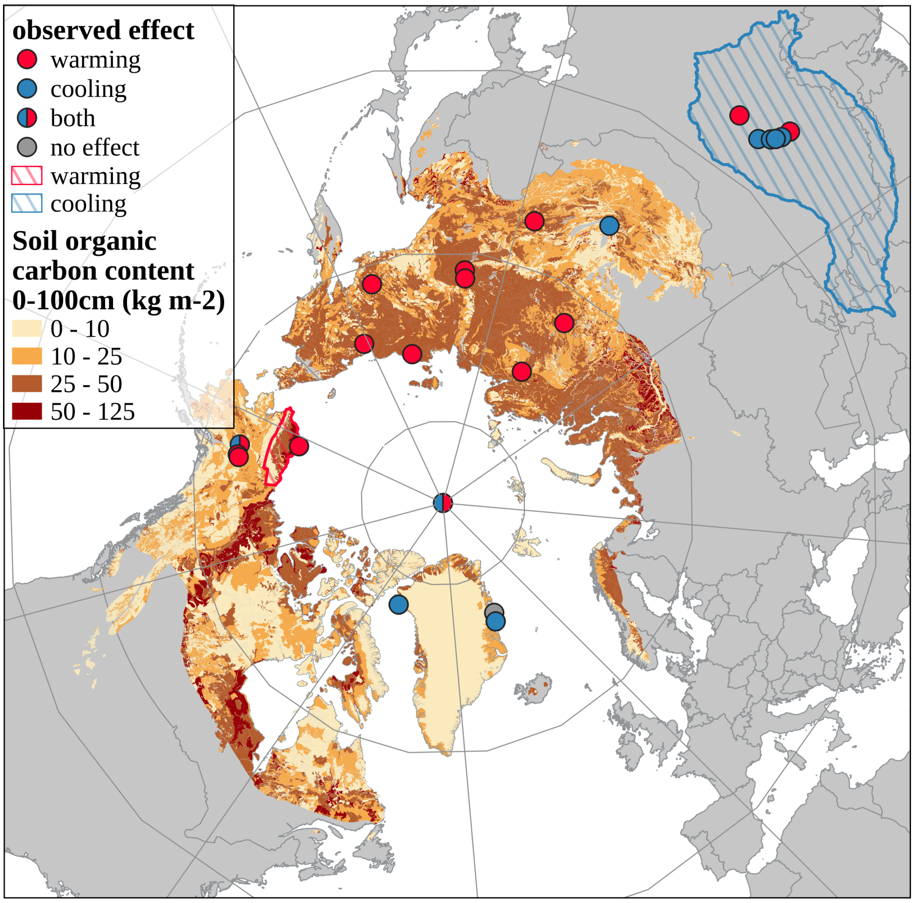

Figure S10: Map of carbon content in the upper 100 cm in the Arctic and Subarctic (without alpine permafrost) based on Hugelius et al. [20]. *Low* is defined as an ice content between 0 and 10%, *medium* is defined as 10–20%, and *high* is defined as >20%. The circles represent the observed effects of summer rainfall on soil temperatures analogous to Figure 1 in the main text. Basemap data has been retrieved from `https://thematicmapping.org/`.

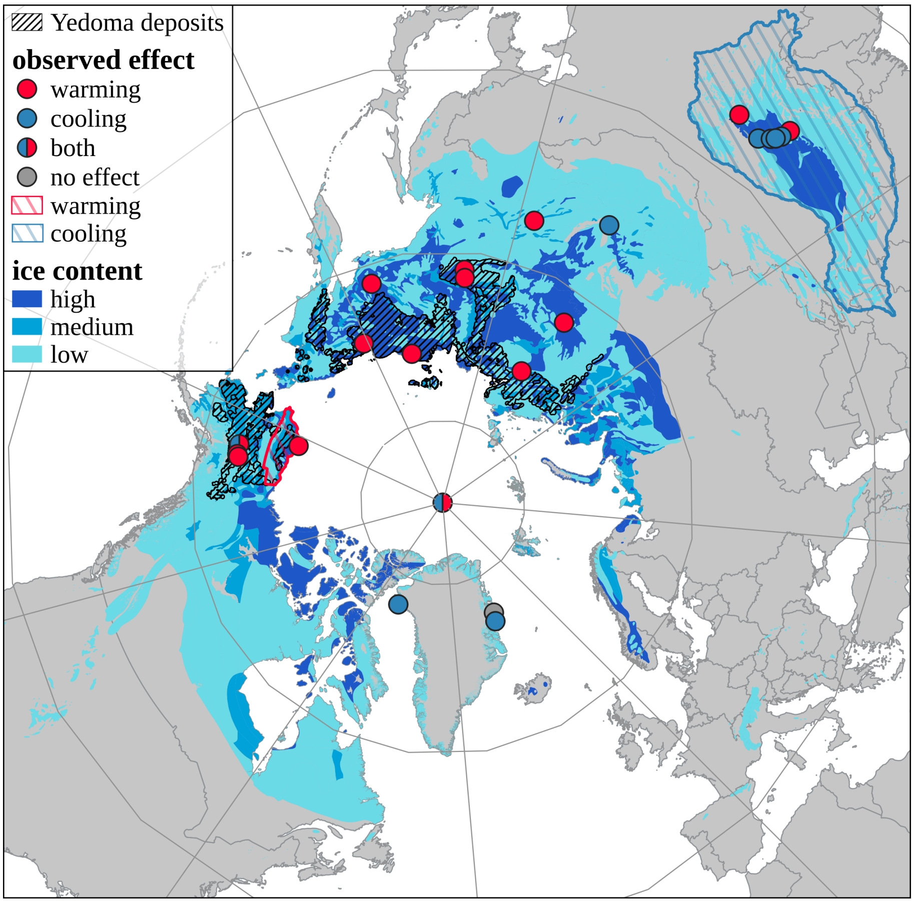

Figure S11: Map of ice content in permafrost landscapes based on Brown et al. [[21]] and location of Yedoma deposits based on [22]. *Low* is defined as an ice content between 0 and 10%, *medium* is defined as 10–20% and *high* is defined as >20%. The circles represent the observed effects of summer rainfall on soil temperatures analogous to Figure 1 in the main text. Basemap data has been retrieved from `https://thematicmapping.org/`.

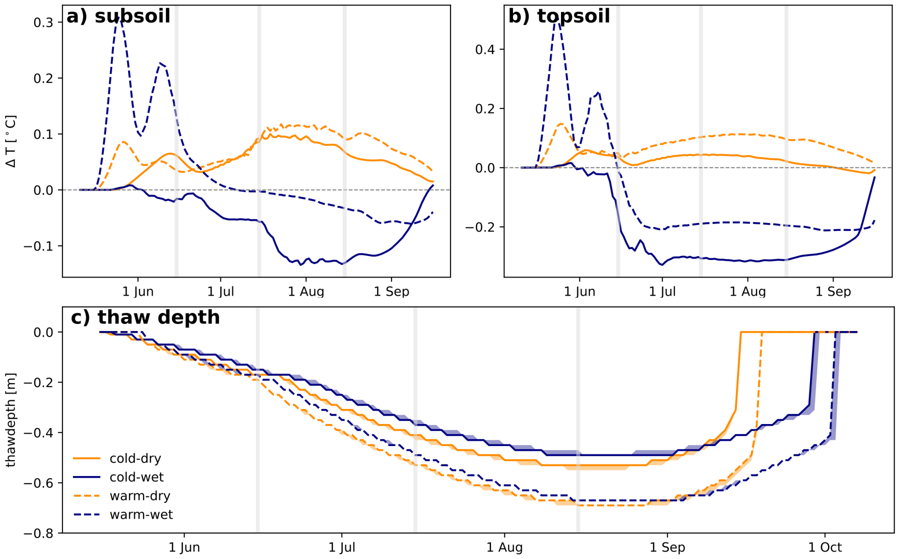

Figure S12: Temperature difference in the uniformly increased precipitation scenario between the heavy rainfall (HR) case and ref. case in **(a)** the subsoil and **(b)** the topsoil as well as thaw depth progression **(c)**. Differences are displayed as HR case minus reference case. Daily values are averaged over a 7-day window.

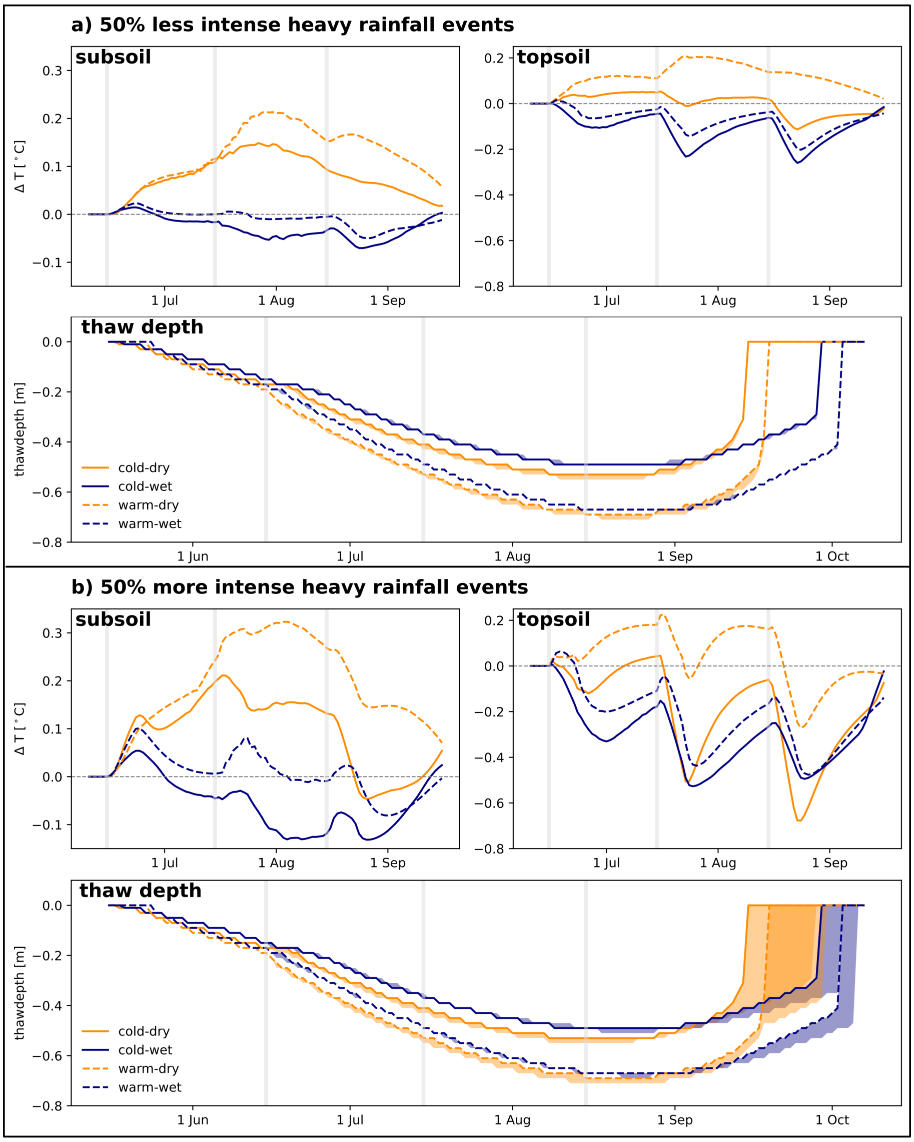

Figure S13: Sensitivity analysis on temperature difference between the heavy rainfall (HR) case and the reference case from the first heavy rain event until the end of the thawing season in the subsoil and the topsoil as well as thaw depth development for (**a**) a scenario with 50% less intense heavy rainfall events as compared to the original scenario and (**b**) a scenario with 50% more intense heavy rainfall events in comparison to the intensity of heavy rain events in the original scenario. Differences are displayed as HR case minus reference case. Daily values are smoothed over a 7-day window. Grey vertical bars indicate the timing of the simulated heavy rain events in each summer month (June, July, August).

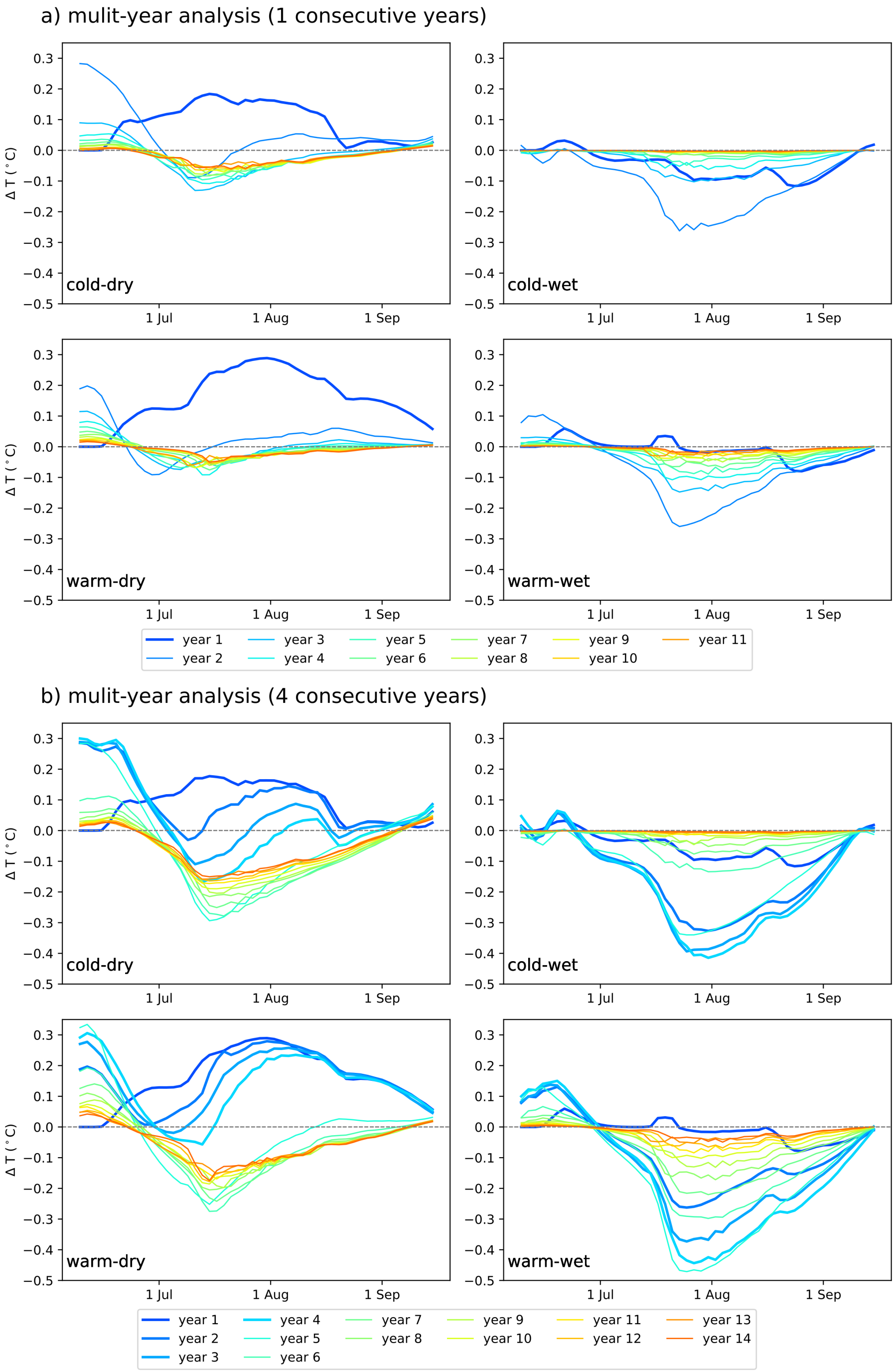

Figure S14: Multiyear subsoil temperature differences over the summer period based on (**a**) a single year of heavy summer rainfall events followed by ten years of baseline summer rainfall and (**b**) four consecutive years of heavy summer rainfall followed by ten years of baseline heavy rainfall. Values are shown for every other day and averaged over a 3-time-step-window.

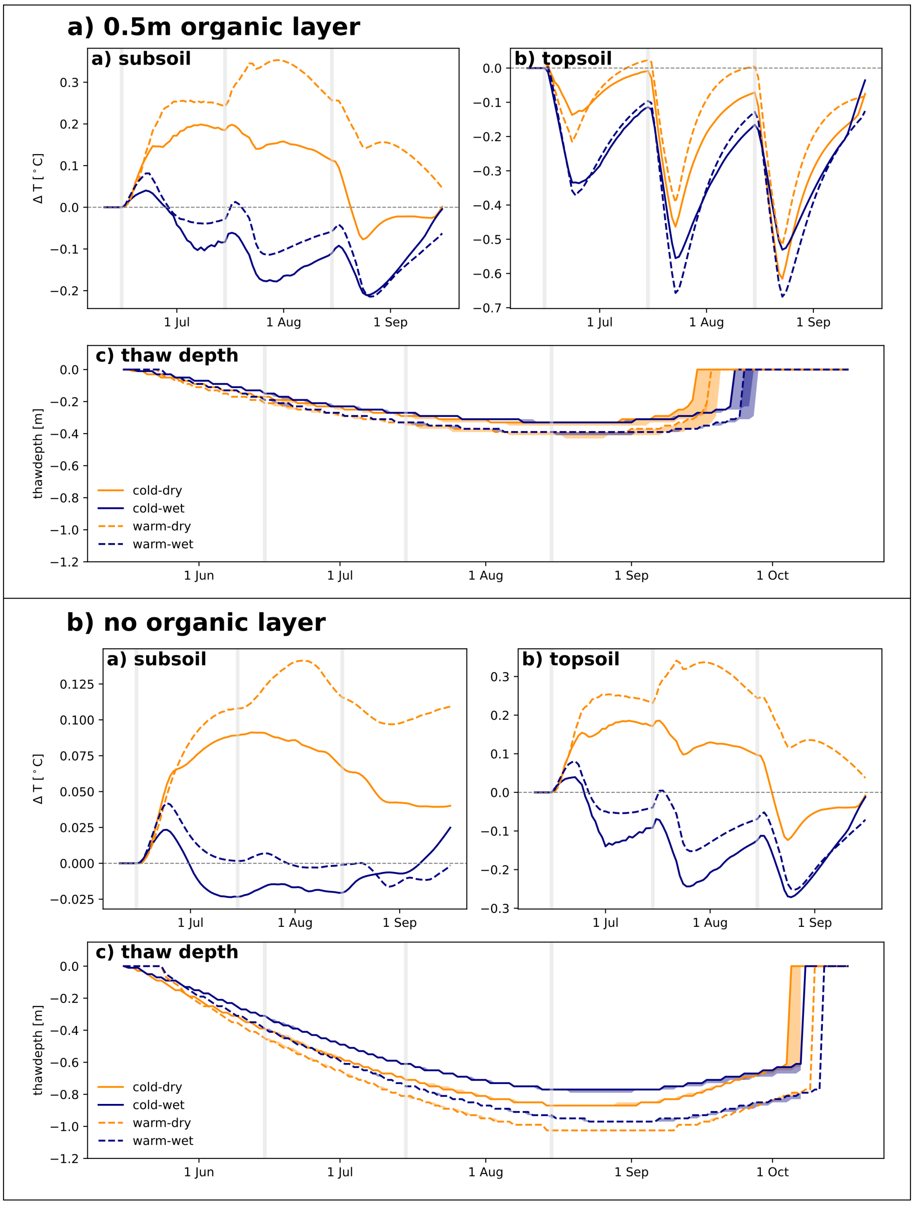

Figure S15: Effect of heavy summer rainfall events on soil temperature differences between the HR case and the ref. case in a scenario with **(a)** a thicker organic layer of 0.5 m and **(b)** no organic layer in the subsoil and topsoil as well as thaw depth progression for each scenario. Grey bars indicate the timing of the heavy rainfall events and daily temperature differences values are averaged over a 7-day window.

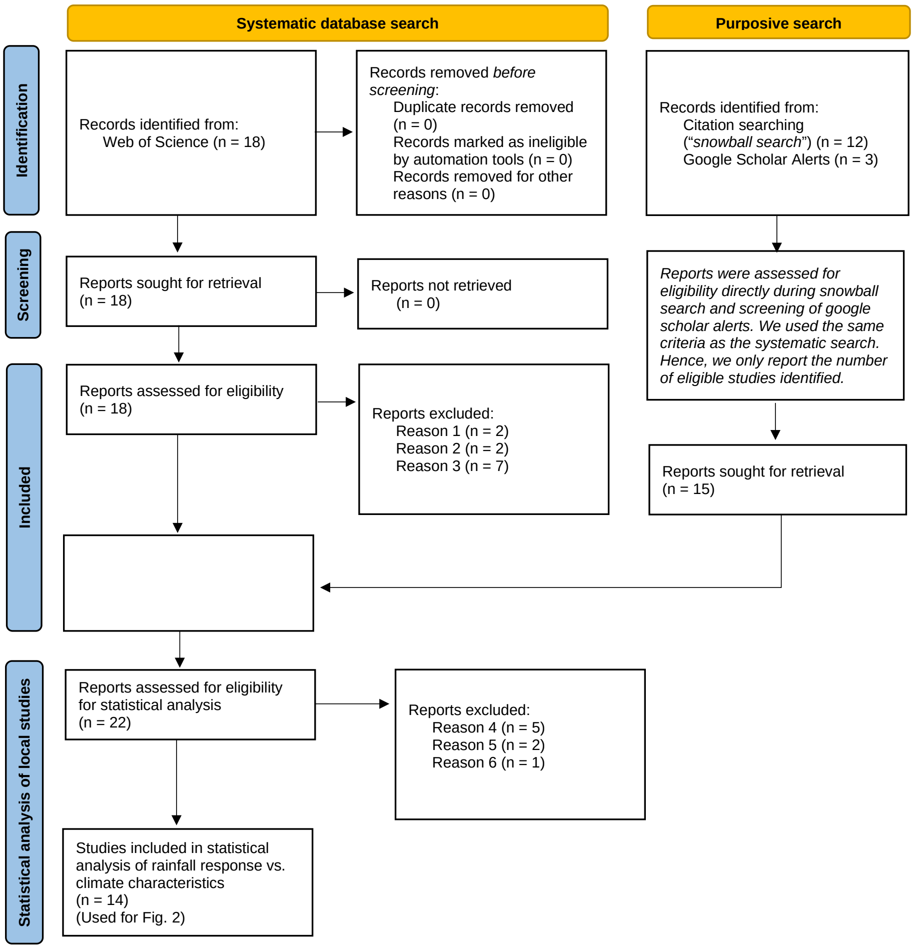

Figure S16: PRISMA diagram of hybrid literature search strategy adopted in this study. Based on Page et al. [[23]]. Reason 1) study was outside the domain (ecosystems underlain by permafrost). Reason 2) study did not consider experimental increases in rainfall, modeled increases in rainfall or natural annual variability in rainfall. Reason 3) study did not report either soil temperature, thaw depth or active layer thickness for heavy rainfall vs. control scenarios or treatments, or under natural annual variation in rainfall. Reason 4) study reported modeled effects only. Reason 5) study reported effects of variable or intervention other than summer rainfall. Reason 6) Study only reports next-year effects of late summer heavy rainfall.

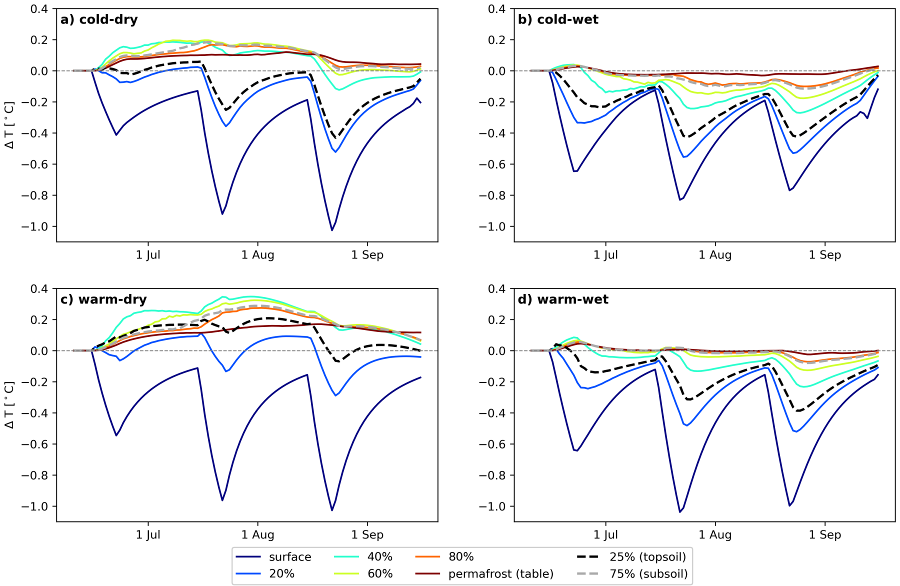

Figure S17: Temperatures differences between the reference and heavy rainfall (HR) case from the first heavy rain event until the end of the thawing season in the (**a**) the cold-dry, (**b**) the cold-wet, (**c**) the warm-dry, and (**d**) the warm-wet case at different relative depths in relation to the maximum active layer thickness ranging from from 0% (surface) to 100% (permafrost table) as well as our chosen depths for topsoil (25%) and subsoil (75%). Daily values are averaged over a 7-day window.

5 Supplementary Tables

Table S1: Summary of literature on the topic of the impact of summer precipitation on the thermal regime of the active layer. A very brief summary of the main effects as well as governing processes in each study are given.

**Author**	**Year**	**Study area**	**Study period**	**Main method**	**rainfall effect**	**Main process**	**Comments**
Zhang et al.	2001	Irkutsk, Russia	1880–2001	Observations	Cooling	Evaporation	Non-permafrost
Illeris et al.	2003	Zackenberg, Greenland	1996–1999	Observations	Topsoil cooling	None given	Precipitation effect not focus of the study
Sullivan et al.	2008	Pituffik, Greenland	2004–2005	Observations	Cooling	None given, but evaporation used to explain negligible differences in soil moisture after irrigation	Cooling effect was not statistically significant
Wu and Zhang	2008	Tibetan Plateau	1996–2006	Observations	Cooling	Evaporation	
Lopez C. et al.	2010	Yakutsk	2004	Observation	Warming	Advective heat transport	
Iijima et al.	2010	Central Lena River Basin	2003–2007	Observations	Warming	Increased thermal conductivity, heat capacity	
Christiansen et al.	2012	Zackenberg, Greenland	2009	Observations	No effect	No effect observed	Only one warm season investigated
Zhu et al.	2017	Tibetan Plateau	2006–2014	Observations	Topsoil: cooling, deeper: warming	Evaporation and heat advection downwards	
Göckede et al.	2017	Siberia	2013–2015	Observations	Topsoil: cooling, subsoil: warming	Differences in thermal conductivity and heat capacity	
Grant et al.	2017	Barrow, Alaska	1980- 2015	Modeling	Warming	Increased thermal conductivity	Focus on soil water content in general, not P specifically
Li et al.	2019	Tibetan Plateau	2013	Modeling and observations	Warming	Heat advection	
Neumann et al.	2019	Alaska	2014–2016	Observation	Warming	Lateral and vertical heat advection	

Table S1: Continuation.

**Author**	**Year**	**Study area**	**Study period**	**Main method**	**rainfall effect**	**Main process**	**Comments**
Karjalainen et al.	2019	Northern hemisphere	2000–2014	Observations	P $\leq$ 250 mm: warming, P $>$ 250 mm: cooling	Increased thermal conductivity (warming) and evaporation and heat capacity (cooling)	
Luo et al.	2020	Tibetan Plateau	2014–2016	Observations	Extreme P: warming, overall: cooling	Extreme P: heat conduction, overall: evaporation	
Douglas et al.	2020	Fairbanks, Alaska	2013–2017	Observations	Warming	Sensible heat flux	
Mekonnen et al.	2021	North Slope, Alaska	1979–2100	Modeling	Warming	Increased heat conduction and advection	
Clayton et al.	2021	Alaska	2013–2018	Observations	Topsoil: warming, subsoil: cooling	Top: thermal conductivity, depth: latent heat requirements	Only considering volumetric water content, not P
Zhang et al.	2021	Tibetan Plateau	2012–2014	Observations and modeling	Cooling	Decreased soil heat flux	
Zhirkov et al.	2021	Yakutsk	2014–2016	Observations and modeling	Irrig. experiment: same summer warming, modeling: warming	Field: none given, modeling: heat conduction	
Zhou et al.	2021	Tibetan Plateau	2013	Observations and modeling	Cooling	Increased latent heat flux, decreased sensible and soil heat flux	
Zhang et al.	2021	Tibetan Plateau	1983–2012	Modeling	Cooling	Increased evaporation, changes in thermal conductivity, latent heat requirements, and heat capacity	
Magnússon et al.	2022	Siberian tundra	2018-2020	Observations and modeling	Warming	Increased heat advection, increased thermal conductivity and heat capacity	Slight topsoil cooling also observed

Table S2: Additional information on study design and reported effect extent, depths (where available), and metric (T: ground temperature, TD: thaw depth, ALT: active layer thickness) throughout the studies used in this work. If different effects where observed in different depths, all are listed. If the effect extent is not explicitly quantified, we label the reported effect as "Not quantified". Based on the depth described in each study, we defined topsoil depths as depth $\leq$ 10 cm and subsoils as depths $>$ 10 cm. We further distinguish between study types that report results of irrigation experiments (irrig. exp.), model results (model) or results based on natural variability (nat var.). In the case of Göckede et al. [5], a drainage experiment forms the basis of the observed effect.

**Study**	**Response class**	**Reported effect**	**Obs. type and depth(s)**	**Rainfall event magnitude**	**Type of study**
Zhang et al. [24]	Cooling	-4°C	T: 40 cm	Attributed to 1mm/yr increase in summer rainfall (1882–1995, 368mm long term average)	nat. var.
Illeris et al. [25]	Topsoil cooling	-1.1°Cand -0.9°C	T: 2 cm and 4 cm	+100% relative to avg. P	irrig. exp.
Sullivan et al. [26]	Cooling	-0.5–0°C	T: 2 cm	+50% relative to avg. P	irrig. exp.
Wu and Zhang [14]	Cooling	Not quantified	T: 100 cm	Variable trends among subsites	nat. var.
Lopez C. et al. [27]	warming	Soil thawing rate increased by 2 cm/day	TD	+120 mm relative to summer P	irirg. exp.
Iijima et al. [7]	Warming	Not quantified	T: 40 and 120 cm	Variable heavy rainfall events among subsites	nat. var.
Christiansen et al. [28]	no effect	Not quantified	T2 and 5 cm	+100% relative to avg. P	irrig. exp.
Zhu et al. [29]	Topsoil: cooling<br>Subsoil: warming	Topsoil: -0.17– -0.2°C<br>subsoil: 0.06–0.09°C	T: Top: 2–10 cm<br>Sub: 105-175 cm	Individual rainfall events<br>12-22 mm, multiple summers	nat. var.
Göckede et al. [5]	Topsoil: cooling<br>Subsoil: warming	Topsoil: up to -10°C<br>Subsoil: up to 1°C	T: Top: 5 cm<br>Sub: 35 cm	Drainage experiment; no rainfall treatment	drainage experiment
Grant et al. [11]	Warming	Not quantified	ALT	Increase of 1.9 mm/yr (1981–2015)	model
Li et al. [12]	Warming	8% and 9% increase in surface heat flux	surface heat fluxes	25% and +50% relative to baseline 19.36 mm rainfall on July 3rd	model
Neumann et al. [30]	Warming	Not quantified	T: subsoil (>75 cm)	Two years with 378 and 345 mm rainfall, compared to a baseline (283 mm/year)	nat. var.
Karjalainen et al. [10]	Both	Not quantified	ALT	Panarctic variability in annual rainfall	nat. var.
Luo et al. [31]	Warming	0.3–0.5°C	T: 160–250 cm	One natural heavy rainfall event (44 mm)	nat. var.
Douglas et al. [32]	Warming	+0.7 cm thaw per cm of additional rain	TD	Two summers with 330 mm and 340 mm, (baseline: 185 mm)	nat. var.
Mekonnen et al. [9]	Warming	1.1°C and +39 cm in ALT	T:0-100 cm, ALT	Model simulations with and without advective heat of rain	model
Clayton et al. [6]	Topsoil: warming<br>Subsoil: cooling	Not quantified	ALT	Natural gradients in topsoil moisture contents	nat. var.
Zhang et al. [33]	Cooling	-3°C	T: 5 cm	One natural heavy rainfall event (19.4 mm)	nat. var.
Zhang et al. [35]	Cooling	35 cm shallower ATL	ALT	100 mm increase in summer precipitation	model
Zhirkov et al. [34]	Warming	Not quantified	T: 0–40 cm, TD	+200% relative to summer P	irrig. exp.
Zhou et al. [13]	Cooling	-1.6°C	T: 5 cm	Ambient rainfall (290.9 mm) and 25% and 50% rainfall increase	model
Magnússon et al. [4]	Top: cooling<br>Sub: Warming	Top: -1°C<br>Sub: 0.4°C	T: 5 cm<br>T: 20 cm, TD	100 mm (+120% relative to avg summer rainfall)	irrig. exp.

Table S3: Literature studies used for statistical evaluation.

Site	Reference	Reported Effect	Longitude	Latitude
Pituffik Peninsula, Greenland	Sullivan et al. 2008	cooling	-68.5667	76.55
Zackenberg, Greenland	Illeris et al. 2003	cooling	-21	74.3
Zackenberg, Greenland	Christiansen et al. 2012	no effect	-20	75.3
Tibet Highway, Qinghai-Tibet Plateau	Wu and Zhang 2008[1]	cooling	92.2	34
HAYR, Qinghai-Tibet Plateau	Luo et al. 2020	warming	97.56996	35.0161
Irkutsk, Russian	Zhang et al. 2001	cooling	104	52
Indigirka Lowlands, Russia	Magnússon et al. 2022	warming	147.9	71.5
Fairbanks, Alaska	Neumann et al. 2019	warming	-148.3	64.7
Fairbanks, Alaska	Douglas et al. 2020	warming	-147.667	64.876
Tanggula, Qinghai-Tibet Plateau	Zhu et al. 2017	cooling	91.81875	32.72667
Beiluhe, Qinghai-Tibet Plateau	Zhang et al. 2021	cooling	92.56	34.51
Yakutsk, Russia	Zhirkov et al. 2021	warming	129.63	62.08
Yakutsk, Russia	Lopez C. et al. 2010	warming	129.6167	62.25

[1)] This datapoint is split up into two points: North and South of the Tanggula mountain range to account for different prevailing climatic conditions within the large spatial extent of the field observations.

**Parameter**	**Unit**	**Soil layer**	**Value**
van Genuchten alpha	Pa^{-1}	organic<br>mineral	0.0005<br>0.00033
van Genuchten m	-	organic<br>mineral	0.19<br>0.248
Porosity	-	organic<br>mineral	0.85<br>0.5
Permeability	m^2	organic<br>mineral	5e-11<br>1e-13
Density	$kg\,m^{-3}$	organic<br>mineral	700<br>2170
Dry<br>thermal conductivity	$W\,m^{-1}\,K^{-1}$	organic<br>mineral	0.1<br>0.3
Saturated<br>thermal conductivity	$W\,m^{-1}\,K^{-1}$	organic<br>mineral	0.67<br>1.7

Table S4: Model parameters used to define the organic soil layer (0 – -0.2 m) and the mineral soil layer (-0.2 – -40 m) in ATS. The values are chosen based on averages of previous studies such as Schuh et al. [36], Jafarov et al. [37], Jan et al. [38], Hamm and Frampton [39]. The values are not site calibrated due to the generic nature of the study and instead are used as representative for organic and mineral soils.

Climate	Topsoil depth	Subsoil depth	Maximum AL depth
warm-dry	-17.25 cm	-51.75 cm	-69 cm
cold-dry	-13.25 cm	-39.75 cm	-53 cm
warm-wet	-16.75 cm	-50.25 cm	-67 cm
cold-wet	-12.25 cm	-36.75 cm	-49 cm

Table S5: Actual depth of top- and subsoil model observation points expressed in the centroid of the corresponding nearest mesh cell.

Supplementary References

[1] David A. Cook. Systematic and Nonsystematic Reviews: Choosing an Approach. In Debra Nestel, Joshua Hui, Kevin Kunkler, Mark W. Scerbo, and Aaron W. Calhoun, editors, *Healthcare Simulation Research*, pages 55–60. Springer International Publishing, Cham, 2019. ISBN 978-3-030-26836-7 978-3-030-26837-4. doi: 10.1007/978-3-030-26837-4_8. URL http://link.springer.com/10.1007/978-3-030-26837-4_8.

[2] Alessandro Liberati, Douglas G. Altman, Jennifer Tetzlaff, Cynthia Mulrow, Peter C. Gøtzsche, John P. A. Ioannidis, Mike Clarke, P. J. Devereaux, Jos Kleijnen, and David Moher. The PRISMA Statement for Reporting Systematic Reviews and Meta-Analyses of Studies That Evaluate Health Care Interventions: Explanation and Elaboration. *PLoS Medicine*, 6(7):e1000100, July 2009. ISSN 1549-1676. doi: 10.1371/journal.pmed.1000100. URL https://dx.plos.org/10.1371/journal.pmed.1000100.

[3] Claes Wohlin, Marcos Kalinowski, Katia Romero Felizardo, and Emilia Mendes. Successful combination of database search and snowballing for identification of primary studies in systematic literature studies. *Information and Software Technology*, 147:106908, July 2022. ISSN 09505849. doi: 10.1016/j.infsof.2022.106908. URL https://linkinghub.elsevier.com/retrieve/pii/S0950584922000659.

[4] Rúna I. Magnússon, Alexandra Hamm, Sergey V. Karsanaev, Juul Limpens, David Kleijn, Andrew Frampton, Trofim C. Maximov, and Monique M. P. D. Heijmans. Extremely wet summer events enhance permafrost thaw for multiple years in Siberian tundra. *Nature Communications*, 13(1):1556, December 2022. ISSN 2041-1723. doi: 10.1038/s41467-022-29248-x. URL https://www.nature.com/articles/s41467-022-29248-x.

[5] Mathias Göckede, Fanny Kittler, Min Jung Kwon, Ina Burjack, Martin Heimann, Olaf Kolle, Nikita Zimov, and Sergey Zimov. Shifted energy fluxes, increased Bowen ratios, and reduced thaw depths linked with drainage-induced changes in permafrost ecosystem structure. *The Cryosphere*, 11(6):2975–2996, December 2017. ISSN 1994-0424. doi: 10.5194/tc-11-2975-2017. URL https://tc.copernicus.org/articles/11/2975/2017/.

[6] Leah K Clayton, Kevin Schaefer, Michael J Battaglia, Laura Bourgeau-Chavez, Jingyi Chen, Richard H Chen, Albert C. Chen, Kazem Bakian-Dogaheh, Sarah Grelik, Elchin Jafarov, Lin Liu, Roger John Michaelides, Mahta Moghaddam, Andrew Parsekian, Adrian V Rocha, Sean R Schaefer, Taylor Sullivan, Alireza Tabatabaeenejad, Kang Wang, Cathy J Wilson, Howard A. Zebker, Tingjun Zhang, and Yuhuan Zhao. Active layer thickness as a function of soil water content. *Environmental Research Letters*, 16(5), April 2021. ISSN 1748-9326. doi: 10.1088/1748-9326/abfa4c. URL https://iopscience.iop.org/article/10.1088/1748-9326/abfa4c.

[7] Yoshihiro Iijima, Alexander N. Fedorov, Hotaek Park, Kazuyoshi Suzuki, Hironori Yabuki, Trofim C. Maximov, and Tetsuo Ohata. Abrupt increases in soil temperatures following increased precipitation in a permafrost region, central Lena River basin, Russia. *Permafrost and Periglacial Processes*, 21(1):30–41, January 2010. ISSN 10456740, 10991530. doi: 10.1002/ppp.662. URL http://doi.wiley.com/10.1002/ppp.662.

[8] Brad J. Bushman and Morgan C. Wang. Vote-counting procedures in meta-analysis. In *The handbook of research synthesis and meta-analysis, 2nd ed.*, pages 207–220. Russell Sage Foundation, New York, NY, US, 2009. ISBN 978-0-87154-163-5 (Hardcover).

[9] Zelalem A Mekonnen, William J Riley, Robert F Grant, and Vladimir E Romanovsky. Changes in precipitation and air temperature contribute comparably to permafrost degradation in a warmer climate. *Environmental Research Letters*, 16(2):024008, February 2021. ISSN 1748-9326. doi: 10.1088/1748-9326/abc444. URL https://iopscience.iop.org/article/10.1088/1748-9326/abc444.

[10] Olli Karjalainen, Miska Luoto, Juha Aalto, and Jan Hjort. New insights into the environmental factors controlling the ground thermal regime across the Northern Hemisphere: a comparison between permafrost and non-permafrost areas. *The Cryosphere*, 13(2):693–707, February 2019. ISSN 1994-0424. doi: 10.5194/tc-13-693-2019. URL https://tc.copernicus.org/articles/13/693/2019/.

[11] R. F. Grant, Z. A. Mekonnen, W. J. Riley, H. M. Wainwright, D. Graham, and M. S. Torn. Mathematical Modelling of Arctic Polygonal Tundra with *Ecosys* : 1. Microtopography Determines How Active Layer Depths Respond to Changes in Temperature and Precipitation: Active Layer Depth in Polygonal Tundra. *Journal of Geophysical Research: Biogeosciences*, 122(12):3161–3173, December 2017. ISSN 21698953. doi: 10.1002/2017JG004035. URL `http://doi.wiley.com/10.1002/2017JG004035`.

[12] De-sheng Li, Zhi Wen, Qian-gong Cheng, Ai-guo Xing, Ming-li Zhang, and An-yuan Li. Thermal dynamics of the permafrost active layer under increased precipitation at the Qinghai-Tibet Plateau. *Journal of Mountain Science*, 16(2):309–322, February 2019. ISSN 1672-6316, 1993-0321. doi: 10.1007/s11629-018-5153-5. URL `http://link.springer.com/10.1007/s11629-018-5153-5`.

[13] Zhi-xiong Zhou, Feng-xi Zhou, Ming-li Zhang, Bing-bing Lei, and Zhao Ma. Effect of increasing rainfall on the thermal—moisture dynamics of permafrost active layer in the central Qinghai—Tibet Plateau. *Journal of Mountain Science*, 18(11):2929–2945, November 2021. ISSN 1672-6316, 1993-0321. doi: 10.1007/s11629-021-6707-5. URL `https://link.springer.com/10.1007/s11629-021-6707-5`.

[14] Qingbai Wu and Tingjun Zhang. Recent permafrost warming on the Qinghai-Tibetan Plateau. *Journal of Geophysical Research*, 113(D13):D13108, July 2008. ISSN 0148-0227. doi: 10.1029/2007JD009539. URL `http://doi.wiley.com/10.1029/2007JD009539`.

[15] Hans Hersbach, Bill Bell, Paul Berrisford, Shoji Hirahara, András Horányi, Joaquín Muñoz-Sabater, Julien Nicolas, Carole Peubey, Raluca Radu, Dinand Schepers, Adrian Simmons, Cornel Soci, Saleh Abdalla, Xavier Abellan, Gianpaolo Balsamo, Peter Bechtold, Gionata Biavati, Jean Bidlot, Massimo Bonavita, Giovanna Chiara, Per Dahlgren, Dick Dee, Michail Diamantakis, Rossana Dragani, Johannes Flemming, Richard Forbes, Manuel Fuentes, Alan Geer, Leo Haimberger, Sean Healy, Robin J. Hogan, Elías Hólm, Marta Janisková, Sarah Keeley, Patrick Laloyaux, Philippe Lopez, Cristina Lupu, Gabor Radnoti, Patricia Rosnay, Iryna Rozum, Freja Vamborg, Sebastien Villaume, and Jean-Noël Thépaut. The ERA5 global reanalysis. *Quarterly Journal of the Royal Meteorological Society*, 146(730):1999–2049, July 2020. ISSN 0035-9009, 1477-870X. doi: 10.1002/qj.3803. URL `https://onlinelibrary.wiley.com/doi/10.1002/qj.3803`.

[16] Horace R. Byers, Harry Moses, and Patrick J. Harney. Measurement of Rain Temperature. *Journal of Meteorology*, 6(1):51–55, February 1949. ISSN 0095-9634, 0095-9634. doi: 10.1175/1520-0469(1949)006<0051:MORT>2.0.CO;2. URL `http://journals.ametsoc.org/doi/10.1175/1520-0469(1949)006<0051:MORT>2.0.CO;2`.

[17] Scott L. Painter and Satish Karra. Constitutive Model for Unfrozen Water Content in Subfreezing Unsaturated Soils. *Vadose Zone Journal*, 13(4):vzj2013.04.0071, April 2014. ISSN 15391663. doi: 10.2136/vzj2013.04.0071. URL `http://doi.wiley.com/10.2136/vzj2013.04.0071`.

[18] R. Bintanja, K. van der Wiel, E. C. van der Linden, J. Reusen, L. Bogerd, F. Krikken, and F. M. Selten. Strong future increases in Arctic precipitation variability linked to poleward moisture transport. *Science Advances*, 6(7):eaax6869, February 2020. ISSN 2375-2548. doi: 10.1126/sciadv.aax6869. URL `https://www.science.org/doi/10.1126/sciadv.aax6869`.

[19] Elchin Jafarov and Kevin Schaefer. The importance of a surface organic layer in simulating permafrost thermal and carbon dynamics. *The Cryosphere*, 10(1):465–475, March 2016. ISSN 1994-0424. doi: 10.5194/tc-10-465-2016. URL `https://tc.copernicus.org/articles/10/465/2016/`.

[20] G. Hugelius, J. G. Bockheim, P. Camill, B. Elberling, G. Grosse, J. W. Harden, K. Johnson, T. Jorgenson, C. D. Koven, P. Kuhry, G. Michaelson, U. Mishra, J. Palmtag, C.-L. Ping, J. O'Donnell, L. Schirrmeister, E. A. G. Schuur, Y. Sheng, L. C. Smith, J. Strauss, and Z. Yu. A new data set for estimating organic carbon storage to 3 m depth in soils of the northern circumpolar permafrost region. *Earth System Science Data*, 5(2):393–402, December 2013. ISSN 1866-3516. doi: 10.5194/essd-5-393-2013. URL `https://essd.copernicus.org/articles/5/393/2013/`.

[21] Jerry Brown, J. Heginbottom, Oscar Ferrians, and E.S. Melnikov. Circum-Arctic Map of Permafrost and Ground-Ice Conditions, Version 2, 2002. URL `https://nsidc.org/data/GGD318/versions/2`. type: dataset.

[22] Jens Strauss, Sebastian Laboor, Lutz Schirrmeister, Alexander N. Fedorov, Daniel Fortier, Duane Froese, Matthias Fuchs, Frank Günther, Mikhail Grigoriev, Jennifer Harden, Gustaf Hugelius, Loeka L. Jongejans, Mikhail Kanevskiy, Alexander Kholodov, Viktor Kunitsky, Gleb Kraev, Anatoly Lozhkin, Elizaveta Rivkina, Yuri Shur, Christine Siegert, Valentin Spektor, Irina Streletskaya, Mathias Ulrich, Sergey Vartanyan, Alexandra Veremeeva, Katey Walter Anthony, Sebastian Wetterich, Nikita Zimov, and Guido Grosse. Circum-Arctic Map of the Yedoma Permafrost Domain. *Frontiers in Earth Science*, 9:758360, October 2021. ISSN 2296-6463. doi: 10.3389/feart.2021.758360. URL `https://www.frontiersin.org/articles/10.3389/feart.2021.758360/full`.

[23] Matthew J Page, Joanne E McKenzie, Patrick M Bossuyt, Isabelle Boutron, Tammy C Hoffmann, Cynthia D Mulrow, Larissa Shamseer, Jennifer M Tetzlaff, Elie A Akl, Sue E Brennan, Roger Chou, Julie Glanville, Jeremy M Grimshaw, Asbjørn Hróbjartsson, Manoj M Lalu, Tianjing Li, Elizabeth W Loder, Evan Mayo-Wilson, Steve McDonald, Luke A McGuinness, Lesley A Stewart, James Thomas, Andrea C Tricco, Vivian A Welch, Penny Whiting, and David Moher. The PRISMA 2020 statement: an updated guideline for reporting systematic reviews. *BMJ*, page n71, March 2021. ISSN 1756-1833. doi: 10.1136/bmj.n71. URL `https://www.bmj.com/lookup/doi/10.1136/bmj.n71`.

[24] T Zhang, Roger G Barry, D Gilichinsky, S S Bykhovets, V A Sorokovikov, and Jingping Ye. An Amplified Signal of Climatic Change in Soil Temperatures during the Last Century at Irkutsk, Russia. *Climatic Change*, page 36, 2001.

[25] L Illeris, A Michelsen, and S Jonasson. Soil plus root respiration and microbial biomass following water, nitrogen, and phosphorus application at a high arctic semi desert. *Biogeochemistry*, page 15, 2003.

[26] Patrick F. Sullivan, Jeffrey M. Welker, Heidi Steltzer, Ronald S. Sletten, Birgit Hagedorn, Seth J. T. Arens, and Jennifer L. Horwath. Energy and water additions give rise to simple responses in plant canopy and soil microclimates of a high arctic ecosystem. *Journal of Geophysical Research*, 113(G3):G03S08, May 2008. ISSN 0148-0227. doi: 10.1029/2007JG000477. URL `http://doi.wiley.com/10.1029/2007JG000477`.

[27] M. L. Lopez C., T. Shirota, G. Iwahana, T. Koide, T. C. Maximov, M. Fukuda, and H. Saito. Effect of increased rainfall on water dynamics of larch (*Larix cajanderi*) forest in permafrost regions, Russia: an irrigation experiment. *Journal of Forest Research*, 15(6):365–373, December 2010. ISSN 1341-6979, 1610-7403. doi: 10.1007/s10310-010-0196-7. URL `https://www.tandfonline.com/doi/full/10.1007/s10310-010-0196-7`.

[28] Casper T. Christiansen, Sarah H. Svendsen, Niels M. Schmidt, and Anders Michelsen. High arctic heath soil respiration and biogeochemical dynamics during summer and autumn freeze-in – effects of long-term enhanced water and nutrient supply. *Global Change Biology*, 18(10):3224–3236, October 2012. ISSN 1354-1013, 1365-2486. doi: 10.1111/j.1365-2486.2012.02770.x. URL `https://onlinelibrary.wiley.com/doi/10.1111/j.1365-2486.2012.02770.x`.

[29] Xiaofan Zhu, Tonghua Wu, Ren Li, Changwei Xie, Guojie Hu, Yanhui Qin, Weihua Wang, Junming Hao, Shuhua Yang, Jie Ni, and Cheng Yang. Impacts of Summer Extreme Precipitation Events on the Hydrothermal Dynamics of the Active Layer in the Tanggula Permafrost Region on the Qinghai-Tibetan Plateau: Impacts of Precipitation on Active Layer. *Journal of Geophysical Research: Atmospheres*, 122(21):11,549–11,567, November 2017. ISSN 2169897X. doi: 10.1002/2017JD026736. URL `http://doi.wiley.com/10.1002/2017JD026736`.

[30] Rebecca B. Neumann, Colby J. Moorberg, Jessica D. Lundquist, Jesse C. Turner, Mark P. Waldrop, Jack W. McFarland, Eugenie S. Euskirchen, Colin W. Edgar, and Merritt R. Turetsky. Warming Effects of Spring Rainfall Increase Methane Emissions From Thawing Permafrost. *Geophysical Research Letters*, 46(3):

1393–1401, February 2019. ISSN 0094-8276, 1944-8007. doi: 10.1029/2018GL081274. URL https://onlinelibrary.wiley.com/doi/abs/10.1029/2018GL081274.

[31] Dongliang Luo, Huijun Jin, Victor F. Bense, Xiaoying Jin, and Xiaoying Li. Hydrothermal processes of near-surface warm permafrost in response to strong precipitation events in the Headwater Area of the Yellow River, Tibetan Plateau. *Geoderma*, 376:114531, October 2020. ISSN 00167061. doi: 10.1016/j.geoderma.2020.114531. URL https://linkinghub.elsevier.com/retrieve/pii/S0016706120306704.

[32] Thomas A. Douglas, Merritt R. Turetsky, and Charles D. Koven. Increased rainfall stimulates permafrost thaw across a variety of Interior Alaskan boreal ecosystems. *npj Climate and Atmospheric Science*, 3(1): 28, December 2020. ISSN 2397-3722. doi: 10.1038/s41612-020-0130-4. URL http://www.nature.com/articles/s41612-020-0130-4.

[33] Mingli Zhang, Zhi Wen, Desheng Li, Yaling Chou, Zhixiong Zhou, Fengxi Zhou, and Bingbing Lei. Impact process and mechanism of summertime rainfall on thermal–moisture regime of active layer in permafrost regions of central Qinghai–Tibet Plateau. *Science of The Total Environment*, 796:148970, November 2021. ISSN 00489697. doi: 10.1016/j.scitotenv.2021.148970. URL https://linkinghub.elsevier.com/retrieve/pii/S0048969721040420.

[34] Aleksandr Zhirkov, Petr Permyakov, Zhi Wen, and Anatolii Kirillin. Influence of Rainfall Changes on the Temperature Regime of Permafrost in Central Yakutia. *Land*, 10(11):1230, November 2021. ISSN 2073-445X. doi: 10.3390/land10111230. URL https://www.mdpi.com/2073-445X/10/11/1230.

[35] Guofei Zhang, Zhuotong Nan, Lin Zhao, Yijia Liang, and Guodong Cheng. Qinghai-Tibet Plateau wetting reduces permafrost thermal responses to climate warming. *Earth and Planetary Science Letters*, 562:116858, May 2021. ISSN 0012821X. doi: 10.1016/j.epsl.2021.116858. URL https://linkinghub.elsevier.com/retrieve/pii/S0012821X21001175.

[36] Carina Schuh, Andrew Frampton, and Hanne Hvidtfeldt Christiansen. Soil moisture redistribution and its effect on inter-annual active layer temperature and thickness variations in a dry loess terrace in Adventdalen, Svalbard. *The Cryosphere*, 11(1):635–651, February 2017. ISSN 1994-0424. doi: 10.5194/tc-11-635-2017. URL https://www.the-cryosphere.net/11/635/2017/.

[37] Elchin E Jafarov, Ethan T Coon, Dylan R Harp, Cathy J Wilson, Scott L Painter, Adam L Atchley, and Vladimir E Romanovsky. Modeling the role of preferential snow accumulation in through talik development and hillslope groundwater flow in a transitional permafrost landscape. *Environmental Research Letters*, 13(10): 105006, October 2018. ISSN 1748-9326. doi: 10.1088/1748-9326/aadd30. URL http://stacks.iop.org/1748-9326/13/i=10/a=105006?key=crossref.ea8d38a9a41cbb120144acdd5d1d4d37.

[38] Ahmad Jan, Ethan T. Coon, and Scott L. Painter. Evaluating integrated surface/subsurface permafrost thermal hydrology models in ATS (v0.88) against observations from a polygonal tundra site. *Geoscientific Model Development*, 13(5):2259–2276, May 2020. ISSN 1991-9603. doi: 10.5194/gmd-13-2259-2020. URL https://www.geosci-model-dev.net/13/2259/2020/.

[39] Alexandra Hamm and Andrew Frampton. Impact of lateral groundwater flow on hydrothermal conditions of the active layer in a high arctic hillslope setting. *The Cryosphere*, April 2021. doi: 10.5194/tc-2021-60. URL https://tc.copernicus.org/preprints/tc-2021-60/.
